# Epicardial left ventricular leads via minimally invasive technique: a role of steroid eluting leads

**DOI:** 10.1186/s13019-017-0659-4

**Published:** 2017-11-08

**Authors:** Etem Caliskan, Florian Fischer, Felix Schoenrath, Maximilian Y. Emmert, Francesco Maisano, Volkmar Falk, Christoph T. Starck, Tomas Holubec

**Affiliations:** 1 Clinic for Cardiovascular Surgery, University Hospital Zurich, University of Zurich, Zurich, Switzerland; 2 Department of Cardiothoracic and Vascular Surgery, German Heart Institute Berlin, Berlin, Germany; 3 Department of Cardiac Surgery, Kerckhoff Heart and Lung Center, 61231 Bad Nauheim, Germany

**Keywords:** Cardiac resynchronization therapy, Left ventricular lead, Steroid eluting lead, Non-steroid eluting lead, Minimally invasive

## Abstract

**Background:**

We retrospectively assessed two types of sutureless screw-in left ventricular (LV) leads (steroid eluting vs. non-steroid eluting) in cardiac resynchronization therapy (CRT) implantation with regards to their electrical performance.

**Methods:**

Between March 2008 and May 2014 an epicardial LV lead was implanted in 32 patients after failed transvenous LV lead placement using a left-sided lateral minithoracotomy or video-assisted thoracoscopy (mean age 64 ± 9 years). Patients were divided into two groups according to the type of implanted lead. Steroid eluting (SE) group: 21 patients (Myodex™ 1084 T; St. Jude Medical) and non-steroid eluting (NSE) group: 11 patients (MyoPore® 511,212; Greatbatch Medical).

**Results:**

All epicardial leads could be placed successfully, without any intraoperative complications or mortality. With regard to the implanted lead following results were observed: sensing (mV): SE 8.8 ± 6.1 vs. NSE 10.1 ± 5.3 (*p* = 0.380); pacing threshold (V@0.5 ms): SE 1.0 ± 0.5 vs. NSE 0.9 ± 0.5 (*p* = 0.668); impedance (ohms): SE 687 ± 236 vs. NSE 790 ± 331 (*p* = 0.162). At the follow-up (2.6 ± 1.9 years) the following results were seen: sensing (mV): SE 8.7 ± 5.0 vs. NSE 11.2 ± 6.6 (*p* = 0.241), pacing threshold (V@0.5 ms): SE 1.4 ± 0.5 vs. NSE 1.0 ± 0.3 (*p* = 0.035), impedance (ohms): SE 381 ± 95 vs. NSE 434 ± 88 (*p* = 0.129).

**Conclusions:**

Based on the results no strong differences have been found between the both types of epicardial LV leads (steroid eluting vs. non-steroid eluting) in CRT implantation in short- and midterm.

## Background

The prevalence and incidence of heart failure has continuously increased in US and Europe during the last decades [1]. Despite the advances in the optimal medical treatment, strategies and therapies for medically refractory symptomatic advanced heart failure have emerged, including cardiac resynchronization therapy. Patients with New York Heart Association (NYHA) class III or IV heart failure, who have left ventricular ejection fraction (EF) of 35% or less, a sinus rhythm with a QRS duration of ≥120 ms and a left bundle branch block (LBBB) QRS morphology or a QRS duration of ≥150 ms irrespective of QRS morphology are according to the current guidelines eligible to receive a cardiac resynchronization therapy (CRT) [2, 3]. With a considerable number of CRT implantations worldwide, the implantation success rate has reached up to 90% with increasing experience of implantation. The transvenous approach represents the “gold standard” in implantation technique for CRT. However, in case of failed transvenous coronary sinus lead placement or lead extraction due to infection or lead failure, epicardial left ventricular (LV) lead placement is required. Additionally, in patients undergoing cardiac interventions with open chest surgery and present indication for CRT, the implantation of the left ventricular lead can also be performed epicardially. Despite few comparative studies on the different surgical LV leads, little is known about the short- and long-term performance of the different sutureless epicardial lead types.

The aim of the study was to investigate the differences between two types of sutureless screw-in LV leads (steroid eluting vs. non-steroid eluting) in CRT implantation with regards to the electrical performance on the short- and mid-term.

## Methods

### Study population and clinical data

We identified and retrospectively evaluated 32 consecutive patients who underwent sutureless epicardial left ventricular lead implantation either via left lateral minithoracotomy, or video-assisted thoracoscopy at our institution between March 2008 and May 2014. Preoperative variables and demographics are summarized in Table 1. An intraoperative conversion or extension due to complications was not necessary. Patients were divided into two groups: steroid eluting LV lead (SE) group and non-steroid eluting (NSE) LV lead group.

**Table 1:** The preoperative data according to the type of left ventricular lead

	SE group (*n* = 21)	NSE group (n = 11)	p-value
Age, mean ± SD, years	67 ± 7	63 ± 11	0.158
Female gender	4 (19.1%)	4 (36.4%)	0.397
DCM	14 (66.7%)	7 (63.6%)	0.864
ICM	7 (33.3%)	4 (36.4%)	1.000
EF, mean ± SD, %	25 ± 7	24 ± 8	0.613
NYHA, mean ± SD	3 ± 1	3 ± 1	0.969
QRS duration, mean ± SD, ms	156 ± 25	155 ± 28	0.271

SE group – patients with steroid eluting left ventricular lead; NSE group – patients with non-steroid eluting left ventricular lead. DCM – dilative cardiomyopathy; EF – ejection fraction; ICM – ischemic cardiomyopathy; NYHA – New York Heart Association functional class; QRS – electrocardiogram QRS complex; SD – standard deviation

Perioperative and follow-up data was noted for operative time (minute), LV ejection fraction (%), clinical NYHA class and the following electrical parameters: sensing of underlying rhythm in millivolts (mV), pacing lead impedance in ohms (Ω) and pacing thresholds measured with a pulse width of 0.5 ms in volts (V @ 0.5 ms).

Follow-up information was collected from our database or medical records of referring cardiologists.

Data collection was performed prospectively. This study was approved by local ethics committee (Ref. KEK-ZH-Nr. 2014-0017) and the informed consent was obtained from each patient.

### Surgical technique

LV lead implantation of either steroid eluting (Myodex™ 1084 T, St. Jude Medical, Inc., St. Paul, MN, USA) or non-steroid eluting (MyoPore® 511,212, Greatbatch Medical, Clarence, NY, USA) bipolar sutureless screw-in leads were carried out through minithoracotomy or video-assisted thoracoscopy.

#### Minithoracotomy

Epicardial left ventricular lead implantation via left lateral minithoracotomy was performed in standard fashion as previously described in general anesthesia and double-lumen endotracheal intubation for selective lung ventilation [4]. Briefly, anterolateral minithoracotomy was performed in the fourth or fifth intercostal space. The phrenic nerve was identified and the pericardium was opened posteriorly, pericardial stay sutures were placed for cardiac exposure, if necessary. After identification of the optimal implantation site, sutureless leads were placed on the designated location. Electrical parameters were then measured and if necessary the leads repositioned.

#### Video-assisted thoracoscopy

Briefly, the procedure was carried out in general anesthesia with the patient in supine position and selective lung ventilation [4]. Three 10–12 mm ports were inserted in the left hemithorax for endoscopic instruments and scope. Under identification of the phrenic nerve the pericardium was opened posteriorly and optimal implantation site was selected. The screw-in leads were then placed. Electrical parameters were then measured and if necessary the leads repositioned.

### Statistical analysis

Continuous and discrete variables are presented as mean ± standard deviation (SD) and medians and range for data not normally distributed. They were compared using a two-sample *t*-test or Mann-Whitney test, where appropriate. Categorical and ordinal data are presented as number and percentage of observations. They were compared using Pearson’s Chi-squared test or Fischer’s exact test, where appropriate. The probability of freedom from event was calculated according to the Kaplan-Meier method. Freedom-from-event curves were compared by log-rank test. A *p*-value <0.05 was considered to indicate statistical significance. Statistical analysis was performed using IBM SPSS Statistics, Version 22 (IBM Corp., Armonk, NY, USA).

## Results

A total of 32 patients with mean age of 65 ± 9 years (38–78 years) underwent a surgical implantation of epicardial steroid and non-steroid eluting sutureless LV leads. Dilated cardiomyopathy was present in 66% and ischemic cardiomyopathy in 34% of patients with a mean QRS length of 156 ± 25 ms. Median NYHA class was III and mean EF was 25 ± 7%. Preoperative variables and demographics are summarized in Table 1.

Twenty-one patients received steroid eluting leads (SE group) and 11 patients non-steroid eluting leads (NSE group). Twenty-eight patients underwent epicardial LV lead implantation via left minithoracotomy and in 4 patients the video-assisted thoracoscopy was performed. All but two patients have undergone previous failed transvenous LV lead placement. All epicardial leads were placed successfully, without any intraoperative complication. Median operative time was 90 (45–165) minutes in the SE group and 105 (60–215) minutes in the NSE group (*p* = 0.411) and mean length of hospital stay was 4 (1–16) days in the SE group and 6 (2–33) days in the NSE group (*p* = 0.223), respectively. No statistical significance was observed with regards to sensing, pacing threshold, impedance, and length of hospital stay between the groups. Operative data are shown in Table 2.

**Table 2:** The operative data according to the type of left ventricular lead

	SE group (*n* = 21)	NSE group (*n* = 11)	p-value
Operative time, mean ± SD, min	110 ± 49	116 ± 57	0.411
Length of hospital stay, mean ± SD, days	6.4 ± 6.0	7.5 ± 7.3	0.223
30d-Mortality	0	0	1.000
Sensing, mean ± SD, mV	8.8 ± 6.1	8.4 ± 5.2	0.380
Pacing threshold, mean ± SD, V@0.5 ms	1.0 ± 0.5	1.0 ± 0.7	0.668
Impedance, mean ± SD, ohms	687 ± 236	707 ± 302	0.162

SE group – patients with steroid eluting left ventricular lead; NSE group – patients with non-steroid eluting left ventricular lead. SD – standard deviation

**Fig. 1:**
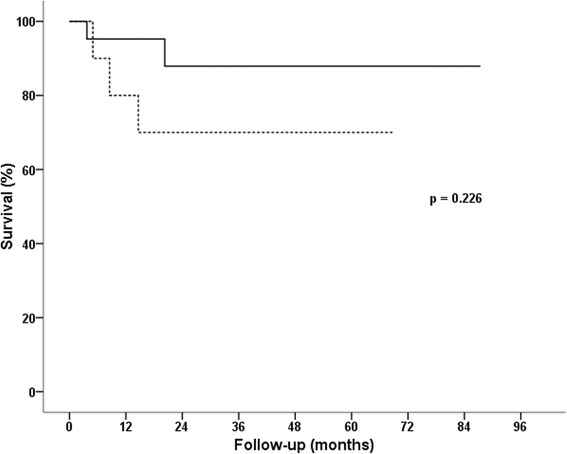
Kaplan-Meier curves showing survival in patients after sutureless left ventricular lead implantation (steroid eluting – solid line vs. non-steroid eluting – dashed line) in cardiac resynchronization therapy

The 30-days mortality was 0%. The mean follow-up was 2.6 ± 1.9 years (median 2.3 years, range 0.2–7.3 years). Four late deaths (one in SE group and three in NSE group) occurred during the follow-up (Fig. 1), two for cardiac reasons and two of unknown cause. One patient had a lead associated endocarditis in which the CRT system including the epicardial LV lead was explanted. A subsequent reimplantation of a new device was not undertaken due to patient’s refusal. One CRT system was explanted 23 months after the implantation in a patient who was listed for heart transplantation after receiving a donor heart. During the follow-up, significant increase of LV-EF and improved NYHA functional class was apparent with no difference between both groups (Table 3). Sensing and pacing threshold slightly increased throughout the whole cohort, but without statistically significant difference between the groups. However, values for impedance significantly decreased, again without being different within the SE and NSE groups (Table 3). The 4-year estimated survival was 88 ± 8% and 70 ± 15% for SE and NSE group (*p* = 0.226; Fig. 1), respectively.

**Table 3:** The follow-up data according to the type of left ventricular lead

	SE group (*n* = 21)	NSE group (*n* = 11)	p-value
EF, mean ± SD, %	36.9 ± 11.8	38.1 ± 14.4	0.561
NYHA, mean ± SD	2.1 ± 0.6	2.1 ± 0.6	1.000
Responder rate	15 (71.4%)	8 (72.7%)	0.652
Mortality	1 (4.8%)	3 (27.2%)	0.226
Sensing, mean ± SD, mV	9.5 ± 5.7	9.6 ± 6.1	0.241
Pacing threshold, mean ± SD, V@0.5 ms	1.3 ± 0.5	1.3 ± 0.6	0.074
Impedance, mean ± SD, ohms	396 ± 94	417 ± 106	0.056

SE group – patients with steroid eluting left ventricular lead; NSE group – patients with non-steroid eluting left ventricular lead. EF – ejection fraction; NYHA – New York Heart Association functional class; SD – standard deviation

## Discussion

The “standard of care” of lead implantation for cardiac resynchronization therapy remains still the less invasive transvenous approach [5]. However, due to conditions limiting the transvenous LV lead placement, a surgical placement of an epicardial LV lead is then required. Several issues may result in failed transvenous implantation of the LV lead and/or requiring even primarily surgical epicardial LV lead placement. As such, anatomical limitations due to occlusion of the subclavian vein or the superior vena cava and abnormal anatomy of the coronary sinus are worth mentioning. Furthermore, lead-related issues such as lead instability with repeated dislodgement, phrenic nerve stimulation despite electrical or physical optimization or systemic conditions such as endocarditis as well as issues due to previous transvenous lead implantation may contribute to failed transvenous LV lead implantation [6].

Epicardial LV lead placement can be performed via left anterolateral minithoracotomy, video-assisted thoracoscopy or with the support of a robotically enhanced telemanipulation system requiring general anesthesia with its associated risks [4]. The surgical approach offers excellent view on the targeted area and LV lead placement takes place under direct visualization. Earlier studies have shown similar outcomes of the surgical approach with regards to endurance and performance of epicardial LV leads [5, 7, 8]. Burger and colleagues assessed aspects of different technical concepts (screw-in vs. suture-on leads) of epicardial LV lead placement and demonstrated an excellent long-term performance and durability [9].

Short- and long-term effects of steroid eluting transvenous and epicardial pacing leads in pediatric and congenital heart disease patients showed to be beneficial [10–12]. However, literature on the performance of steroid eluting vs. non-steroid eluting epicardial LV leads is scarce, particularly in the adult cardiac surgery population.

The present study enrolled 32 patients after implantation of steroid eluting (*n* = 21) and non-steroid eluting (*n* = 11) screw-in LV leads. Sensing and pacing thresholds remained stable over the median follow-up time of 2.6 ± 1.9 years throughout the whole cohort, with low median pacing thresholds of 1.3 V at 0.5 ms and no difference was observed between both groups. These findings are in line with previous reports [11, 13]. Nevertheless, in a study by Kutyifa et al. transvenous steroid eluting leads showed a significantly lower pacing threshold compared to non-steroid eluting transvenous leads [14]. Horenstein and colleagues compared the performance of steroid eluting to non-steroid eluting epicardial leads in a growing pediatric population over a period of 10 years. They concluded that steroid eluting epicardial leads outperformed non-steroid eluting leads by their stable and chronic low threshold over time and subsequent lower energy requirement [13]. The electrode-tissue interface (area between cathode of the lead and epimyocardium) is sought to be one of the critical factors for effective energy delivery to cardiac chambers. By the incorporation of steroid elution by the early 1980’s to this area, reliable and low stimulation threshold pacing became possible by reducing the inflammatory response at the site of implantation [15, 16]. Results of the current study by means of stable pacing and sensing thresholds, lead impedance and good lead survival seem to be consistent with the findings of earlier reports.

The present study has several limitations. Although prospective collection of data was performed, this was a retrospective, non-randomized, observational study with a heterogeneous patient population, and all inherent disadvantages apply. Next, the study is based on a single-center experience. Finally, the number of patients in groups was limited and the follow-up period was relatively short.

## Conclusions

All leads proved to be safe and effective in minimally invasive epicardial left ventricular for cardiac resynchronization therapy. Based on the results of the current study no strong differences have been found between the both types of bipolar sutureless screw-in left ventricular leads (steroid eluting vs. non-steroid eluting) in short- and mid-term period. Further studies with larger number of patients and possibly with randomized controlled design and long-term follow up are needed to further elucidate on this important issue.

## Abbreviations

CRT: Cardiac resynchronization therapy
DCM: Dilative cardiomyopathy
EF: Ejection fraction
ICM: Ischemic cardiomyopathy
LBBB: Left bundle branch block
LV: Left ventricle
ms: Millisecond
mV: Millivolt
NSE: Non-steroid eluting
NYHA: New York Heart Association
QRS: Electrocardiogram QRS complex
SD: Standard deviation
SE: Steroid eluting
V: Volt
Ω: Ohm

## References

[1] Mozaffarian D, Benjamin EJ, Go AS, Arnett DK, Blaha MJ, Cushman M (2015). Heart disease and stroke statistics--2015 update: a report from the American Heart Association. Circulation.

[2] Tracy CM, Epstein AE, Darbar D, Dimarco JP, Dunbar SB, Estes NA (2012). 2012 ACCF/AHA/HRS focused update of the 2008 guidelines for device-based therapy of cardiac rhythm abnormalities: a report of the American College of Cardiology Foundation/American Heart Association task force on practice guidelines. J Am Coll Cardiol.

[3] Brignole M, Auricchio A, Baron-Esquivias G, Bordachar P, Boriani G, Breithardt OA (2013). 2013 ESC guidelines on cardiac pacing and cardiac resynchronization therapy: the task force on cardiac pacing and resynchronization therapy of the European Society of Cardiology (ESC). Developed in collaboration with the European heart rhythm association (EHRA). Eur Heart J.

[4] Navia JL, Atik FA (2005). Minimally invasive surgical alternatives for left ventricle epicardial lead implantation in heart failure patients. Ann Thorac Surg.

[5] Doll N, Piorkowski C, Czesla M, Kallenbach M, Rastan AJ, Arya A (2008). Epicardial versus transvenous left ventricular lead placement in patients receiving cardiac resynchronization therapy: results from a randomized prospective study. Thorac Cardiovasc Surg.

[6] Valls-Bertault V, Mansourati J, Gilard M, Etienne Y, Munier S, Blanc JJ (2001). Adverse events with transvenous left ventricular pacing in patients with severe heart failure: early experience from a single centre. Europace.

[7] Ailawadi G, Lapar DJ, Swenson BR, Maxwell CD, Girotti ME, Bergin JD (2010). Surgically placed left ventricular leads provide similar outcomes to percutaneous leads in patients with failed coronary sinus lead placement. Heart Rhythm.

[8] Mair H, Sachweh J, Meuris B, Nollert G, Schmoeckel M, Schuetz A (2005). Surgical epicardial left ventricular lead versus coronary sinus lead placement in biventricular pacing. Eur J Cardiothorac Surg.

[9] Burger H, Kempfert J, van Linden A, Szalay Z, Schoenburg M, Walther T (2012). Endurance and performance of two different concepts for left ventricular stimulation with bipolar epicardial leads in long-term follow-up. Thorac Cardiovasc Surg.

[10] Moracchini PV, Cornacchia D, Bernasconi M, Tesorieri MC, Fabbri M, Marzegalli M (1999). High impedance low energy pacing leads: long-term results with a very small surface area steroid-eluting lead compared to three conventional electrodes. Pacing and clinical electrophysiology : PACE.

[11] Paech C, Kostelka M, Dahnert I, Flosdorff P, Riede FT, Gebauer RA (2014). Performance of steroid eluting bipolar epicardial leads in pediatric and congenital heart disease patients: 15 years of single center experience. J Cardiothorac Surg.

[12] Tomaske M, Gerritse B, Kretzers L, Pretre R, Dodge-Khatami A, Rahn M (2008). A 12-year experience of bipolar steroid-eluting epicardial pacing leads in children. Ann Thorac Surg.

[13] Horenstein MS, Hakimi M, Walters H, Karpawich PP (2003). Chronic performance of steroid-eluting epicardial leads in a growing pediatric population: a 10-year comparison. Pacing Clin Electrophysiol.

[14] Kutyifa V, Zima E, Molnar L, Kuehne C, Theiss S, Herrmann G (2013). Direct comparison of steroid and non-steroid eluting small surface pacing leads: randomized, multicenter clinical trial. Cardiology journal.

[15] Mond HG, Helland JR, Stokes K, Bornzin GA, McVenes R (2014). The electrode-tissue interface: the revolutionary role of steroid-elution. Pacing Clin Electrophysiol.

[16] Dvorak P, Novak M, Kamaryt P, Slana B, Lipoldova J, Dvorak P (2012). Histological findings around electrodes in pacemaker and implantable cardioverter-defibrillator patients: comparison of steroid-eluting and non-steroid-eluting electrodes. Europace.

